# Traditional Chinese Medicine Syndromes for Essential Hypertension: A Literature Analysis of 13,272 Patients

**DOI:** 10.1155/2014/418206

**Published:** 2014-02-10

**Authors:** Jie Wang, Xingjiang Xiong, Wei Liu

**Affiliations:** Department of Cardiology, Guang'anmen Hospital, China Academy of Chinese Medical Sciences, Beijing 100053, China

## Abstract

*Background*. To simplify traditional Chinese medicine syndrome differentiation and allow researchers to master syndrome differentiation for hypertension, this paper retrospectively studied the literature and analyzed syndrome elements corresponding to hypertension syndromes. *Methods*. Six databases including PubMed, EMBASE, Chinese Bio-Medical Literature Database, Chinese National Knowledge Infrastructure, Chinese Scientific Journal Database, and Wan-fang Data were searched from 1/January/2003 to 30/October/2013. We included all clinical literature testing hypertension syndromes and retrospectively studied the hypertension literature published from 2003 to 2013. Descriptive statistics calculated frequencies and percentages. *Results*. 13,272 patients with essential hypertension were included. Clinical features of hypertension could be attributed to 11 kinds of syndrome factors. Among them, seven syndrome factors were excess, while four syndrome factors were deficient. Syndrome targets were mainly in the liver and related to the kidney and spleen. There were 33 combination syndromes. Frequency of single-factor syndromes was 31.77% and frequency of two-factor syndromes was 62.26%. *Conclusions*. Excess syndrome factors of hypertension patients include yang hyperactivity, blood stasis, phlegm turbidity, internal dampness, and internal fire. Deficient syndrome factors of hypertension patients are yin deficiency and yang deficiency. Yin deficiency with yang hyperactivity, phlegm-dampness retention, and deficiency of both yin and yang were the three most common syndromes in clinical combination.

## 1. Introduction

Hypertension is an important public health issue worldwide because of its high prevalence and concomitant increase in disease risk [1–3]. It has been estimated that 29% of the world's adult population, or approximately 1.56 billion people, will have hypertension by 2025 [4, 5]. Complementary and alternative medicine (CAM) is becoming increasingly popular [6–13] and numerous interventions are regularly recommended to lower elevated blood pressure (BP) [14–17]. Traditional Chinese medicine (TCM), including herbal medicine and acupuncture, is an important component of CAM therapies [18–21]. Hypertension could be improved by insights from TCM and considerable progress has been made in lowering BP by TCM [22–26].

Syndrome differentiation is a diagnostic and treatment method used in TCM [27, 28]. It plays an important role in the therapeutic process and affects the therapeutic result of certain diseases [29–31]. The syndrome is not only the basic unit of TCM theory and syndrome differentiation, but also the bridge to associating disease and formula [32–35]. TCM syndrome, which is different from a disease or symptoms, is the abstraction of a major pathogenesis. Syndromes are identified from a comprehensive analysis of all symptoms and signs (including tongue appearance and pulse feeling) from the four main diagnostic TCM methods: observation, listening, questioning, and pulse analyses [36–40]. However, syndromes are the product of speculation in TCM. Therefore, they depend on medical experience, academic origins, and other factors. Therefore, the concept of syndromes is vague and broad, which makes clinical application difficult. Syndrome elements, which are the minimum units of syndromes, contribute to simplifying syndrome differentiation and understanding TCM syndromes. Each element has specific symptoms.

To simplify TCM syndrome differentiation and enable researchers not familiar with Chinese medicine to master the laws of hypertension syndrome differentiation, this paper retrospectively studied the literature for 13,272 patients with hypertension, published from 2003 to 2013. This study is beneficial to deepening of the understanding of hypertension and providing a basis and reference for clinical treatment using TCM syndrome differentiation.

## 2. Materials and Methods

### 2.1. Database and Search Strategies

Six databases including PubMed, EMBASE, Chinese Bio-Medical Literature Database (CBM), Chinese National Knowledge Infrastructure (CNKI), Chinese Scientific Journal Database (VIP), and Wan-fang Data were searched from 1/January/2003 to 30/October/2013. Databases in Chinese were searched to retrieve the maximum possible number of trials of syndrome differentiation for essential hypertension (EH) because syndrome differentiation is mainly used in China. Ongoing registered clinical trials were searched at the International Clinical Trial Registry by the U.S. National Institutes of Health (http://clinicaltrials.gov/). The following search terms were used individually or combined: “hypertension,” “blood pressure,” “essential hypertension,” “syndrome differentiation,” “vertigo,” “headache,” “parting,” and “traditional Chinese medicine therapy.” The bibliographies of included studies were searched for additional references.

### 2.2. Inclusion and Exclusion Criteria

Systolic blood pressure (SBP) ≥140 mmHg (1 mmHg = 0.133 kPa) and diastolic blood pressure (DBP) ≥90 mmHg from the literature were based on 1999 WHO-ISH Guidelines for the Management of Hypertension (1999 WHO-ISH GMH), 1998 WHO-ISH Guidelines for the Management of Hypertension (1998 WHO-ISH GMH), 2000 WHO-ISH Guidelines for the Management of Hypertension (2000 WHO-ISH GMH), Chinese Guidelines for the Management of Hypertension-2005 (CGMH-2005), China Guidelines on Prevention and Management of High Blood Pressure-2006 (CGPMHBP-2006), and Seventh Report of the Joint National Committee on Prevention, Detection, Evaluation, and Treatment of High Blood Pressure (JNC 7). Syndrome differentiation of TCM diagnosis used the Standard of TCM Diagnosis and Curative Effect of Disease-Syndrome, published by the State Administration of Traditional Chinese Medicine in 1994. Standards of dialectical classification used Clinical Research Guiding Principles of New Medicine of Chinese Herbs revised by the State Food and Drug Administration in 2002. Exclusion criteria were secondary hypertension, gestational hypertension, repeated literature, reviews, and literature with no clear classification.

### 2.3. Classification Criteria of Syndrome Elements

According to the classification criteria of syndrome elements proposed by Wang, statistical analysis was conducted for syndromes included in the cases. The classification criteria of syndrome elements were (1) six-excess external contraction: wind, cold, dampness, dryness, and fire; (2) five endogenous qi: internal wind, internal cold, internal dampness, internal dryness, and internal fire; (3) factors related to gas: qi deficiency, qi stagnation, qi block, qi counterflow, qi fall, and qi collapse; (4) factors related to blood: blood deficiency, blood stasis, blood collapse, blood dryness, and bleeding; (5) factors related to yin and yang: yin deficiency, yang deficiency, yin exuberance, and yang hyperactivity; (6) others: poison, excessive fluid, and phlegm turbidity.

### 2.4. Statistical Methods

Two authors conducted the literature search, study selection, and data extraction independently. Disagreements were resolved by discussion and consensus was met through a third party. SPSS 11.5 statistical software was used for data analyses (Chicago, IL, USA). Descriptive statistics procedures calculated frequency and percentage.

## 3. Results

### 3.1. Description of Included Literature

**Figure 1:**
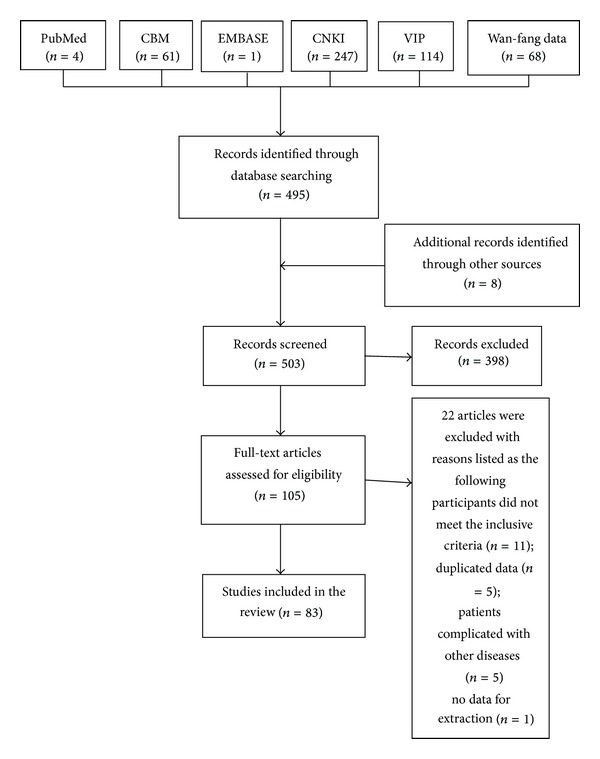
Screening process of articles.

After a primary search of the databases, 503 articles were screened. After reading the titles and abstracts, 398 articles were excluded the reasons included; retrospective study that did not match the included criteria of this review (*n* = 42) and duplicated titles (*n* = 356). The full texts of 83 articles [41–123] were retrieved, and 22 articles were excluded for the following reasons: participants not meeting the inclusion criteria (*n* = 11), duplicated data (*n* = 5), patients having other diseases (*n* = 5), and no data for extraction (*n* = 1). In the end, 83 articles [41–123] were included, and all trials were conducted in China (Figure 1). The characteristics of included trials are listed in Table 1.

Overall, 13,272 patients with essential hypertension were included, with an average of 160 per trial, ranging from 23 to 703. Among them, 7075 were men, accounting for 53.3%, while 6197 were women, accounting for 46.7%. There was a wide range in patient age (18–92 years). Sources of cases included 24 provinces and the number of papers in each region is shown in Table 2.

### 3.2. Extraction of Syndrome Elements of EH

According to the definition of syndrome elements and classification criteria, syndrome elements were obtained and classified from the literature as follows: blood stasis (qi stagnation and blood stasis, qi deficiency with blood stasis, kidney deficiency and blood stasis, stasis blocking channels, phlegm and blood stasis resistance winding); qi stagnation (liver qi stagnation, qi stagnation and blood stasis); phlegm (phlegm turbidity resistance, phlegm-dampness retention); internal fire (intense liver fire, internal harassment of phlegm-heat); internal dampness (spleen deficiency with dampness encumbrance, phlegm-damp retention); internal wind (internal stirring of liver wind, wind-yang interference); qi deficiency (dual deficiency of qi and yin, dual deficiency of qi and blood, and qi deficiency with blood stasis); yang hyperactivity (ascendant hyperactivity of liver yang, yin deficiency with yang hyperactivity); yin deficiency (yin deficiency with yang hyperactivity, liver-kidney yin deficiency, dual deficiency of qi and yin, and deficiency of both yin and yang); yang deficiency (kidney yang deficiency, deficiency of both yin and yang); blood deficiency (dual deficiency of qi and blood). As a result, 13,272 cases of hypertension syndrome were classified as 11 syndrome element types, which cover all cases.

### 3.3. Analysis of Syndrome Elements of EH

**Figure 2:**
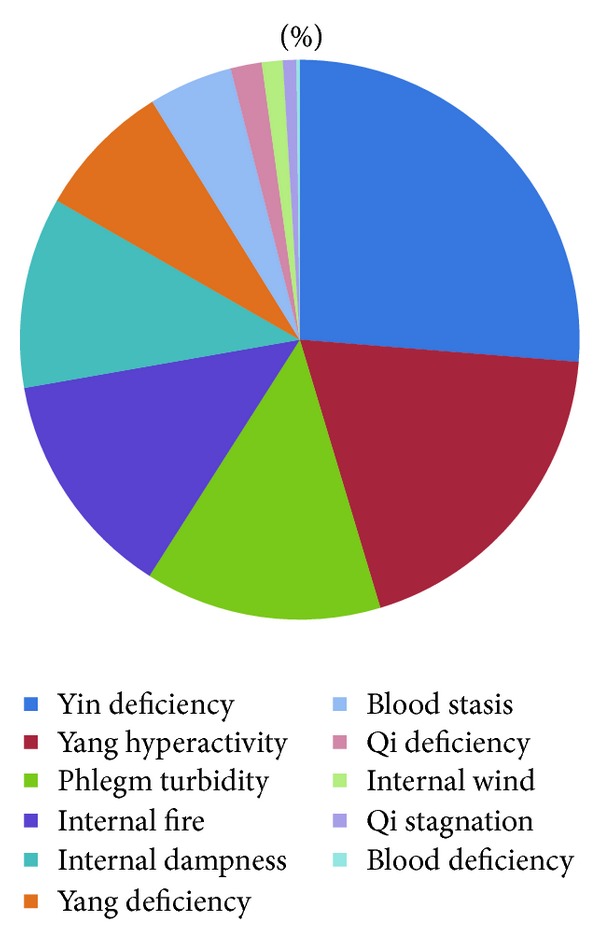
Percentage of syndrome factors.

Syndrome elements of 13,272 patients with hypertension were divided into excessive syndrome elements and deficient syndrome elements (Table 3, Figure 2). The proportions of excessive syndrome elements are yang hyperactivity (19.08%), phlegm turbidity (13.68%), internal fire (13.21%), internal dampness (11.04%), blood stasis (4.86%), internal wind (1.21%), and qi stagnation (0.78%). The proportion of deficient syndrome elements are yin deficiency (26.27%), yang deficiency (7.89%), qi deficiency (1.80%), and blood deficiency (0.18%). Excessive syndrome elements greater than 10% included yang hyperactivity, phlegm turbidity, internal fire, and internal dampness. Deficient syndrome elements greater than 10% included yin deficiency. Yang hyperactivity and yin deficiency were the most common syndrome elements of hypertension.

### 3.4. Targets of Syndrome Elements of EH

The targets of syndrome elements are the disease locations of individual syndrome elements. Disease location of syndrome elements was confirmed according to the five zang-organs and six fu-organs, chi heng fu, and meridians.

As a result, 9091 cases (68.50%) had clear targets of syndrome elements related to liver, kidney, and spleen (Table 4). There were 7789 cases of liver syndromes (85.68%). Among them, there were 2793 cases of internal fire of liver (35.86%), 4033 cases of ascendant hyperactivity of liver yang (51.78%), 543 cases of liver yin deficiency (6.97%), 164 cases of liver qi stagnation (2.11%), and 256 cases of internal stirring of liver wind (3.29%). There were 903 cases of kidney syndromes (9.93%). Among them, there were 879 cases of kidney yin deficiency (97.34%) and 24 cases of kidney yang deficiency (2.66%). There were 399 cases of spleen syndromes (4.39%), all of which were spleen qi deficiency.

### 3.5. Combining Forms of Syndrome Elements of EH

We found that 13,272 cases of hypertension contained 33 syndrome types. According to the definition of syndrome elements, all syndromes were divided into four types: single factor, two-factor, three-factor, and four-factor syndromes. The statistics of the combined forms of syndrome and their frequency (proportion more than 1%) are shown in Table 5. Internal fire is the most common in the single factor group, while yin deficiency with yang hyperactivity is the most common in the two-factor group. From highest to lowest frequency in the two-factor group are phlegm-damp retention, deficiency of both yin and yang, Liver-kidney yin deficiency, dual deficiency of qi and yin, qi stagnation and blood stasis, and qi deficiency with blood stasis. The syndrome, yin deficiency and wind-phlegm, is the most common in the three-factor category. There were no four-factor combinations that reached a frequency of greater than 1%.

## 4. Discussion and Perspectives

### 4.1. Pathogenesis of Hypertension

Syndrome elements are the expression of pathogenesis of a disease [36]. According to the statistical results of syndrome elements, pathogenesis of EH can be summarized as simultaneous insufficiency and excess. Deficiency syndrome included yin deficiency, yang deficiency, qi deficiency, and blood deficiency. Excess syndrome included blood stasis, phlegm turbidity, qi stagnation, yang hyperactivity, internal fire, internal dampness, and internal wind. Among them, yin deficiency was the most common, followed by yang hyperactivity. Other elements, included in descending order, were phlegm turbidity, internal fire, internal dampness, yang deficiency, blood stasis, qi deficiency, and internal wind. The main disease location is the liver, which is closely related to the kidney and spleen.

### 4.2. Characteristics of Combined Syndrome Elements of EH

The combined forms of syndrome elements of hypertension have certain characteristics according to the literature, summarized as follows. (1) The combined forms of syndrome elements of hypertension have three forms, single-factor, two-factor, and three-factor forms. (2) Excess syndromes are more common than deficiency syndromes for single-factor syndromes, with internal fire, yang hyperactivity, blood stasis, and phlegm turbidity as the main syndrome factors. (3) Deficiency syndrome and excess syndrome was the most common two-factor syndrome, followed by excess syndrome and excess syndrome and deficiency syndrome and deficiency syndrome, respectively. (4) Syndrome of yin deficiency and wind-phlegm was the most common three-factor syndrome.

### 4.3. Implications for Instructing Clinical Application

The discovery of distributing characteristics of syndrome elements is conducive to instructing clinical application. Several Chinese herbs and classical formulas can lower BP and improve symptoms according to syndrome differentiation (Table 6). First, when aiming to cure internal fire syndrome, use *Huanglian Jie Du Tang* (detoxicant decoction of* Coptis*) to clear heat and toxins of the liver [35]. Chinese herbs such as *Xiakucao* (*Prunella vulgaris* L.) [123], *Huanglian* (*Coptis chinensis*) [124], *Huangqin* (*Scutellaria baicalensis *Georgi), *Huang-bai* (*Phellodendron *bark), and *Zhizi* (*Gardenia*) [125] can lower BP. Second, when aiming to cure yin deficiency with yang hyperactivity, use *Tianma Gouteng Yin* (decoction of* Gastrodia *and* Uncaria*), a famous prescription noted in *Za Bing Zheng Zhi Xin Yi* (*New Meanings in Syndrome and Therapy of Miscellaneous Diseases*). Chinese herbs such as *Tianma* (*Gastrodia*) [126] and *Gouteng* (*Uncaria*) [127] could suppress liver yang hyperactivity. *Niuxi* (*Achyranthes *root) [128] *and Duzhong* (*Eucommia ulmoides*) [129–131] had antihypertensive effects by nourishing the kidney. Third, when aiming to cure phlegm-damp retention, use *Wuling* powder [132], *Zexie Tang* (decoction of* Alisma*) [133], and *Wendantang jiawei *decoction (modified decoction for clearing away gallbladder heat). In addition, when aiming at wind-phlegm syndrome, use *Banxia Baizhu Tianma Tang* (decoction of *Pinellia ternata, Atractylodes macrocephala,* and *Gastrodia elata*) to calm the liver, strengthen the spleen, remove dampness, and reduce phlegm [35]. Chinese herbs such as *Zexie* (*Alisma*), *Fuling* (*Poria cocos*) [134], *Zhuling* (*Polyporus*) [135], and *Banxia* (*The tuber of pinellia*) [136] could effectively reduce BP as well. Fourthly, to remove blood stasis, use *Xuefu Zhuyu Tang*, a famous classical prescription recorded in *Yi Lin Gai Cuo* (*Correction of the Errors of Medical Works*) by Wang Qingren in the Qing Dynasty. It is effective in removing blood stasis and promoting Qi. Herbs such as *Chishao* (red peony root) [137], *Danshen* (*Salvia miltiorrhiza*) [138], *Yimucao* (*Leonurus japonicus*), and *Chuanxiong* (*Ligusticum chuanxiong *Hort) [139] could also lower BP. When aiming to remove qi stagnation and blood stasis, use herbs to promote qi circulation by taking herbs to remove blood stasis. Herbs that promote qi circulation include* Chaihu* (Chinese thorowax root) [137], *Cangzhu* (*Rhizoma Atractylodis*), and *Zhiqiao* (*Fructus Aurantii*). Finally, deficiency syndromes including liver-kidney yin deficiency, yang deficiency, qi deficiency, and blood deficiency are common in hypertension. When curing liver-kidney yin deficiency, use *Liu Wei Dihuang Wan* (pill of* Rehmannia*) [23]. *Liu Wei Dihuang Wan* was recorded in *Xiaoer Yaozheng Zhijue* (*Pediatric Medicine Card Straight*) by *Qianyi* in the Song Dynasty, and it can replenish liver and kidney yin. When treating yang deficiency, use *Shen qi Wan *(kidney qi pill) to recuperate kidney yang. When aiming to treat qi deficiency, use *Huangqi* (*Astragalus membranaceus*) [140–142] and *Baizhu* (*Rhizoma Atractylodis Macrocephalae*). When aiming to treat blood deficiency, use *Danggui* (*Angelica sinensis*) [143], *Shengdihuang* (*Radix Rehmanniae*), *Chuanxiong* (*Ligusticum chuanxiong *Hort) [144], and *Baishao* (*Radix Paeoniae Rubra*).

In summary, the syndrome elements of hypertension are limited and are combined into syndromes. Single and the combined syndrome elements of hypertension are the basis of syndrome differentiation for EH and the key to the standardization of this syndrome. In this paper, we retrospectively confirmed the validity and reliability of the theory of syndrome elements and the combined forms of syndrome elements of hypertension. This study can provide new ideas and methods for the treatment of hypertension by syndrome differentiation, and has laid a foundation for researching syndrome standardization of hypertension.

## Figures and Tables

**Table 1:** Characteristics of included studies.

Study ID	Sample (M/F)	Age (years)	Diagnosis standard	TCM syndrome differentiation (number of patients)	Region of China
Xu and Chen 2012 [13]	122 (58/64)	60–79	Chinese Guidelines for the Management of Hypertension-2005 (CGMH-2005)	Phlegm and blood stasis resistance winding (39), yin deficiency with yang hyperactivity (44), and idney deficiency (39)	Beijing
Ferreira and Lopes 2011 [14]	448 (243/205)	M: 62.1 ± 10.9 F: 59.3 ± 8.7	Chinese Guidelines for the Management of Hypertension-2005 (CGMH-2005)	Intense liver fire (284), yin deficiency with yang hyperactivity (43), phlegm-damp retention (74), and deficiency of both yin and yang (47)	Jiangsu
Wang et al. 2013 [15]	99 (50/49)	73 ± 6.1	1999 WHO-ISH GMH	Qi stagnation and blood stasis (99)	Guangdong
Lee et al. 2004 [16]	87 (48/39)	M: 62.7 ± 8.3 F: 58.9 ± 7.5	Chinese Guidelines for the Management of Hypertension-2005 (CGMH-2005)	Ascendant hyperactivity of liver yang (32), phlegm-damp retention (27), and qi deficiency with blood stasis (28)	Guangdong
Wang et al. 2013 [17]	140 (83/57)	56.5 ± 9.8	Chinese Guidelines for the Management of Hypertension-2010 (CGMH-2010)	Intense liver fire (28), yin deficiency with yang hyperactivity (39), phlegm-damp retention (45), and deficiency of both yin and yang (28)	Heibei
Wang and Xiong 2012 [18]	76 (38/38)	Not reported	Chinese Guidelines for the Management of Hypertension-2010 (CGMH-2010)	Kidney deficiency and blood stasis (76)	Fujian
Xiong et al. 2013 [19]	395 (228/167)	53 ± 17	Hypertension diagnostic criteria (unclear)	Intense liver fire (54), yin deficiency with yang hyperactivity (177), phlegm-damp retention (62), and deficiency of both yin and yang (102)	Liaoning
Wang et al. 2013 [20]	120 (60/60)	29–62	Hypertension diagnostic criteria (unclear)	Ascendant hyperactivity of liver yang (30), yin deficiency with yang hyperactivity (30), phlegm-damp retention (30), and deficiency of both yin and yang (30)	Hainan
Wang et al. 2013 [21]	184 (83/101)	18–80	Chinese Guidelines for the Management of Hypertension-2005 (CGMH-2005)	Intense liver fire (21), yin deficiency with yang hyperactivity (75), phlegm-damp retention (81), and deficiency of both yin and yang (7)	Jiangsu
Chen 1993 [22]	60 (30/30)	T: 48 ± 8.1 C: 47 ± 6.7	1999 WHO-ISH GMH	Dual deficiency of qi and yin (60)	Guangdong
Wang et al. 2012 [23]	53 (16/37)	40–80	1999 WHO-ISH GMH	Insufficiency of spleen with overabundance of dampness (19), dual deficiency of qi and blood (20), and liver-kidney yin deficiency (14)	Neimenggu
Wang et al. 2013 [24]	112 (83/29)	53.5 ± 11.04	1999 WHO-ISH GMH	Intense liver fire (19), yin deficiency with yang hyperactivity (23), phlegm-damp retention (16), and deficiency of both yin and yang (22)	Jiangxi
Wang and Xiong 2012 [25]	61 (M/F not reported)	T: 57.1 ± 6.16 C: 55.67 ± 6.28	Chinese Guidelines for the Management of Hypertension-2004 (CGMH-2004)	Blood stasis (61)	Guangdong
Chen et al. 2011 [26]	259 (108/151)	65.58 ± 12.17	Chinese Guidelines for the Management of Hypertension-2005 (CGMH-2005)	Intense liver fire (35), yin deficiency with yang hyperactivity (89), phlegm-damp retention (88), and deficiency of both yin and yang (47)	Beijing
Xu and Chen 2011 [27]	81 (53/28)	52.79 ± 12.83	Chinese Guidelines for the Management of Hypertension-2005 (CGMH-2005)	Intense liver fire (42), yin deficiency with yang hyperactivity (18), phlegm-damp retention (14), and deficiency of both yin and yang (7)	Zhejiang
Chen et al. 2012 [28]	183 (85/98)	66.81 ± 8.81	Chinese Guidelines for the Management of Hypertension-2004 (CGMH-2004)	Intense liver fire (28), yin deficiency with yang hyperactivity (53), phlegm-damp retention (57), and deficiency of both yin and yang (45)	Jiangsu
Liu et al. 2011 [29]	89 (45/44)	M: 59.5 ± 10.9 F: 59.3 ± 11.0	Chinese Guidelines for the Management of Hypertension-2005 (CGMH-2005)	Intense liver fire (59), yin deficiency with yang hyperactivity (5), phlegm-damp retention (13), and deficiency of both yin and yang (12)	Jiangsu
Dobos and Tao 2011 [30]	342 (213/129)	M: 59.43 ± 16.76 F: 59.43 ± 11.82	1999 WHO-ISH GMH	Intense liver fire (51), yin deficiency with yang hyperactivity (139), phlegm-damp retention (85), and deficiency of both yin and yang (67)	Guangdong
Xiong et al. 2011 [31]	562 (297/265)	M: 62.1 ± 10.8 F: 58.5 ± 9.1	Chinese Guidelines for the Management of Hypertension-2005 (CGMH-2005)	Intense liver fire (352), yin deficiency with yang hyperactivity (58), phlegm-damp retention (97), and deficiency of both yin and yang (55)	Jiangsu
Wang and Xiong 2012 [32]	398 (199/199)	59.20 ± 9.54	Chinese Guidelines for the Management of Hypertension-2005 (CGMH-2005)	Intense liver fire (88), yin deficiency with yang hyperactivity (196), phlegm-damp retention (89), and deficiency of both yin and yang (25)	Jiangsu
Wang et al. 2013 [33]	178 (81/97)	18–80	Chinese Guidelines for the Management of Hypertension-2005 (CGMH-2005)	Intense liver fire (49), yin deficiency with yang hyperactivity (43), phlegm-damp retention (57), and deficiency of both yin and yang (29)	Shanghai
Tian 2011 [34]	200 (109/91)	30–75	1999 WHO-ISH GMH	Intense liver fire (37), yin deficiency with yang hyperactivity (55), phlegm-damp retention (82), and deficiency of both yin and yang (26)	Tianjin
Wang and Xiong 2012 [35]	120 64/56	T: 62.77 ± 9.18 C: 59.63 ± 8.77	1999 WHO-ISH GMH	Intense liver fire (37), yin deficiency with yang hyperactivity (55), phlegm-damp retention (82), and deficiency of both yin and yang (26)	Hunan
Wang et al. 2012 [36]	494 (264/230)	M: 61.6 ± 10.6 F: 58.3 ± 8.5	Chinese Guidelines for the Management of Hypertension-2005 (CGMH-2005)	Ascendant hyperactivity of liver yang (313), yin deficiency with yang hyperactivity (52), deficiency of both yin and yang (83), and liver-kidney yin deficiency (46)	Jiangsu
Xu and Chen 2008 [37]	150 (M/F not reported)	Not reported	Chinese Guidelines for the Management of Hypertension-2005 (CGMH-2005)	Intense liver fire (29), randomized stagnation of phlegm (53), dual deficiency of qi and yin (30), and stasis blocking channels (38)	Xinjiang
Cheung 2011 [38]	109 (68/41)	65.6 ± 10.6	1999 WHO-ISH GMH	Intense liver fire (19), yin deficiency with yang hyperactivity (18), phlegm-damp retention (34), and deficiency of both yin and yang (38)	Fujian
Xiong et al. 2013 [39]	102 (58/44)	37–85	1999 WHO-ISH GMH	Intense liver fire (18), yin deficiency with yang hyperactivity (31), phlegm-damp retention (23), and deficiency of both yin and yang (30)	Guizhou
Lu et al. 2004 [40]	40 (23/17)	Not reported	1999 WHO-ISH GMH	Blood stasis (40)	Guangdong
Zhao et al. 2012 [41]	60 (41/19)	T: 62.07 ± 8.88 C: 57.3 ± 9.09	1999 WHO-ISH GMH and Chinese Guidelines for the Management of Hypertension-2005 (CGMH-2005)	Blood stasis (60)	Guangdong
Liu et al. 2009 [42]	60 (36/24)	T: 53.87 ± 5.92 C: 52.97 ± 5.40	Chinese Guidelines for the Management of Hypertension-2005 (CGMH-2005)	Phlegm-damp retention (60)	Shanghai
Wang et al. 2012 [43]	82 (49/33)	60–75	1999 WHO-ISH GMH	Liver-kidney yin deficiency (82)	Heilongjiang
Luo et al. 2011 [44]	100 (42/58)	36–81	Hypertension diagnostic criteria (unclear)	Ascendant hyperactivity of liver yang (12), yin deficiency with yang hyperactivity (60), phlegm-damp retention (18), and kidney deficiency (10)	Guangxi
Wang et al. 2012 [45]	80 (48/32)	68.05 ± 5.41	1999 WHO-ISH GMH	Liver-kidney yin deficiency (80)	Guangxi
Wang et al. 2011 [46]	251 (148/103)	55 ± 19	1999 WHO-ISH GMH	Intense liver fire (71), yin deficiency with yang hyperactivity (62), phlegm-damp retention (60), and deficiency of both yin and yang (58)	Liaoning
Bai et al. 2005 [47]	122 (71/51)	T: 44.7 ± 11.6 C: 46.2 ± 9.5	1999 WHO-ISH GMH	Ascendant hyperactivity of liver yang (35), liver-kidney yin deficiency (18), phlegm-damp retention (32), dual deficiency of qi and yin (25), and stasis blocking channels (12)	Hebei
Yang et al. 2005 [48]	80 (41/39)	M: 51.28 ± 6.96 F: 52.71 ± 6.57	1999 WHO-ISH GMH	Ascendant hyperactivity of liver yang (80)	Henan
Xia et al. 2010 [49]	40 (M/F not reported)	T: 55.23 ± 6.01 C: 55.13 ± 6.34	1999 WHO-ISH GMH	Ascendant hyperactivity of liver yang (40)	Gansu
Liu et al. 2003 [50]	60 (43/17)	45–73	Chinese Guidelines for the Management of Hypertension-2009 (CGMH-2009)	Yang hyperactivity (29), phlegm turbidity resistance (31)	Zhejiang
Yin and Liu 2005 [51]	36 (M/F not reported)	40.50 ± 11.51	1999 WHO-ISH GMH	Phlegm-damp retention (36)	Jiangsu
Wu et al. 2010 [52]	90 (41/39)	32–78	1999 WHO-ISH GMH	Ascendant hyperactivity of liver yang and blood stasis (90)	Hebei
Deng 2008 [53]	60 (45/15)	T: 61 ± 4.12 C: 61 ± 4.02	1999 WHO-ISH GMH	Qi deficiency with blood stasis (60)	Hebei
Wu and Xu 2010 [54]	60 (32/28)	Not reported	1999 WHO-ISH GMH	Dual deficiency of qi and yin (60)	Guizhou
Wang et al. 2011 [55]	276 170/106	M: 53.4 ± 21.1 F: 55.6 ± 17.3	1999 WHO-ISH GMH	Wind-yang interference (22), stasis blocking channels (73), yin deficiency with yang hyperactivity (134), and phlegm turbidity resistance (47)	Guangxi
Wu et al. 2010 [56]	156 (79/77)	T: 48 ± 6.9 C: 49 ± 8.2	Hypertension diagnostic criteria (unclear)	Ascendant hyperactivity of liver yang (52), yin deficiency with yang hyperactivity (53), and deficiency of both yin and yang (51)	Zhejiang
Fan and Liu 2010 [57]	395 (203/192)	30–80	Chinese Guidelines for the Management of Hypertension-2005 (CGMH-2005)	Qi deficiency with blood stasis (65), intense liver fire (91), yin deficiency with yang hyperactivity (63), phlegm-damp retention (57), deficiency of both yin and yang (39), and dual deficiency of qi and blood (18)	Beijing
Zhu et al. 2009 [58]	54 (30/24)	61.74 ± 14.89	1999 WHO-ISH GMH	Kidney yang deficiency (24), kidney yin deficiency (30)	Yunnan
Liu et al. 2009 [59]	140 (68/72)	34–79	Chinese Guidelines for the Management of Hypertension-2005 (CGMH-2005)	Intense liver fire (16), yin deficiency with yang hyperactivity (52), phlegm-damp retention (41), and deficiency of both yin and yang (31)	Guangxi
He et al. 2013 [60]	230 (65/165)	43–74	1999 WHO-ISH GMH	Intense liver fire (28), ascendant hyperactivity of liver yang (148), and liver-kidney yin deficiency (54)	Guangdong
Tang et al. 2012 [61]	100 (37/63)	55.1 ± 6.2	1999 WHO-ISH GMH	Intense liver fire (19), yin deficiency with yang hyperactivity (29), deficiency of both yin and yang (20), and liver-kidney yin deficiency (32)	Shanghai
Gong et al. 2010 [62]	120 (60/60)	T: 55.38 ± 8.01 C: 56.80 ± 8.58	1999 WHO-ISH GMH	Intense liver fire (30), yin deficiency with yang hyperactivity (30), phlegm-damp retention (30), and deficiency of both yin and yang (30)	Shandong
Zhang et al. 2005 [63]	60 (32/28)	62.22 ± 6.12	Chinese Guidelines for the Management of Hypertension-2004 (CGMH-2004)	Intense liver fire (8), yin deficiency with yang hyperactivity (28), phlegm-damp retention (14), and deficiency of both yin and yang (10)	Guangxi
Liu et al. 2009 [64]	200 (105/95)	M: 61.88 ± 11.91 F: 63.07 ± 12.45	Chinese Guidelines for the Management of Hypertension-2005 (CGMH-2005)	Intense liver fire (51), yin deficiency with yang hyperactivity (49), phlegm-damp retention (50), and deficiency of both yin and yang (50)	Guangxi
Wang 2012 [65]	200 (103/94)	46.4 ± 15.46	1999 WHO-ISH GMH	Intense liver fire (96), yin deficiency with yang hyperactivity (46), phlegm-damp retention (18), and deficiency of both yin and yang (37)	Shanxi
Yao and Huang 2007 [66]	47 (22/25)	66.00 ± 12.35	Chinese Guidelines for the Management of Hypertension-2004 (CGMH-2004)	Intense liver fire (12), yin deficiency with yang hyperactivity (11), phlegm-damp retention (12), and deficiency of both yin and yang (12)	Tianjin
Guo et al. 2002 [67]	120 (62/58)	T: 63.64 ± 9.22 C: 60.30 ± 3.36	Chinese Guidelines for the Management of Hypertension-2004 (CGMH-2004)	Intense liver fire (30), yin deficiency with yang hyperactivity (30), phlegm-damp retention (30), and deficiency of both yin and yang (30)	Anhui
Zhang et al. 2011 [68]	320 (135/185)	66.40 ± 12.56	2007 WHO-ISH GMH	Intense liver fire (36), yin deficiency with yang hyperactivity (101), phlegm-damp retention (125), and deficiency of both yin and yang (58)	Jiangsu
Liao et al. 2010 [69]	23 (14/9)	T: 65 ± 5 C: 65 ± 8	1999 WHO-ISH GMH	Blood stasis (23)	Fujian
Xiong 2010 [70]	70 (37/33)	53.06 ± 8.62	Hypertension diagnostic criteria (unclear)	Intense liver fire (13), yin deficiency with yang hyperactivity (21), phlegm-damp retention (25), and deficiency of both yin and yang (11)	Heilongjiang
Jiang et al. 2012 [71]	86 (50/36)	36–81	Hypertension diagnostic criteria (unclear)	Yin deficiency with yang hyperactivity (86)	Guangdong
Huang and Wei 2012 [72]	260 (119/141)	65.56 ± 8.42	Chinese Guidelines for the Management of Hypertension-2010 (CGMH-2010)	Intense liver fire (56), yin deficiency with yang hyperactivity (77), phlegm-damp retention (73), and deficiency of both yin and yang (54)	Beijing
Lu 2004 [73]	138 (97/41)	61.84 ± 5.25	1999 WHO-ISH GMH	Intense liver fire (16), yin deficiency with yang hyperactivity (43), phlegm-damp retention (45), and deficiency of both yin and yang (34)	Fujian
Sun and Wang 2005 [74]	703 (382/321)	50–79	Chinese Guidelines for the Management of Hypertension-2005 (CGMH-2005)	Yin deficiency with yang hyperactivity (215), phlegm-damp retention (83), deficiency of both yin and yang (91), ascendant hyperactivity of liver yang (135), liver-kidney yin deficiency (92), yang deficiency (11), qi deficiency (14), dual deficiency of qi and yin (14), blood stasis (11), qi deficiency with blood stasis (3), internal harassment of phlegm-heat (22), internal harassment of phlegm-heat and blood stasis (3), liver-kidney yin deficiency and blood stasis (2), internal harassment of phlegm-heat and qi deficiency (1), deficiency of both yin and yang and internal harassment of phlegm-heat (1), liver-kidney yin deficiency and phlegm-damp retention (1), yin deficiency with yang hyperactivity and blood stasis (1), ascendant hyperactivity of liver yang and internal harassment of phlegm-heat (1), ascendant hyperactivity of liver yang and blood stasis (1), and deficiency of both yin and yang and phlegm-damp retention (1)	Guangdong
Xiang et al. 2012 [75]	125 (75/50)	55–72	Hypertension diagnostic criteria (unclear)	Kidney deficiency and blood stasis (15), internal stirring of liver wind (68), qi deficiency with blood stasis (21), and intermingled phlegm and blood stasis (21)	Guangdong
Zhu 2009 [76]	97 (41/56)	37–79	Hypertension diagnostic criteria (unclear)	Ascendant hyperactivity of liver yang (13), yin deficiency with yang hyperactivity (59), liver-kidney yin deficiency (16), and deficiency of both yin and yang (21)	Shandong
Xu and Wang 2009 [77]	80 (49/31)	40–83	1999 WHO-ISH GMH	Intense liver fire (18), yin deficiency with yang hyperactivity (17), phlegm-damp retention (35), and deficiency of both yin and yang (10)	Xinjiang
Lin and Kang 2012 [78]	69 (37/32)	T: 53.48 ± 10.02 C: 59.20 ± 5.610	Chinese Guidelines for the Management of Hypertension-2005 (CGMH-2005)	Liver-kidney yin deficiency (69)	Zhejiang
Feng et al. 2013 [79]	60 (60 M)	63.0 ± 7.5	Chinese Guidelines for the Management of Hypertension-2005 (CGMH-2005)	Ascendant hyperactivity of liver yang (60)	Fujian
Yu and Xing 2010 [80]	168 (108/60)	T: 58 ± 12 C: 54 ± 12	1999 WHO-ISH GMH	Intense liver fire (54), yin deficiency with yang hyperactivity (45), phlegm-damp retention (36), and deficiency of both yin and yang (33)	Beijing
Qiu et al. 2011 [81]	170 (122/48)	54 ± 11.6	Chinese Guidelines for the Management of Hypertension-2004 (CGMH-2004)	Intense liver fire (43), yin deficiency with yang hyperactivity (40), phlegm-damp retention (38), and deficiency of both yin and yang (49)	Beijing
Wu and Xu 2012 [82]	149 (74/75)	61.22 ± 9.36	Hypertension diagnostic criteria (unclear)	Intense liver fire (48), yin deficiency with yang hyperactivity (32), phlegm-damp retention (49), and deficiency of both yin and yang (20)	Hubei
Fang et al. 2007 [83]	220 (128/92)	34–73	1999 WHO-ISH GMH	Intense liver fire (98), yin deficiency with yang hyperactivity (79), phlegm-damp retention (19), and deficiency of both yin and yang (24)	Gansu
Fang et al. 2003 [84]	229 (113/116)	>35	Hypertension diagnostic criteria (unclear)	Liver-kidney yin deficiency (60), yin deficiency with yang hyperactivity (73), phlegm-damp retention (85), and deficiency of both yin and yang (11)	Hangzhou
Peng and Shi 2010 [85]	122 (57/65)	64.62 ± 8.86	1999 WHO-ISH GMH	Qi deficiency with blood stasis (26), intense liver fire (23), yin deficiency with yang hyperactivity (26), phlegm-damp retention (25), and deficiency of both yin and yang (22)	Anhui
Yang et al. 2004 [86]	151 (110/41)	Not reported	Hypertension diagnostic criteria (unclear)	Ascendant hyperactivity of liver yang (151)	Shandong
Shi et al. 2013 [87]	60 (29/31)	52.6 ± 12.3	Clinical research guiding principles of new medicine of Chinese traditional medicine	Phlegm-damp retention (60)	Zhejiang
Han 2004 [88]	377 (182/195)	20–60	Chinese Guidelines for the Management of Hypertension-2005 (CGMH-2005)	Intense liver fire (108), yin deficiency with yang hyperactivity (70), phlegm-damp retention (154), and deficiency of both yin and yang (45)	Anhui
Shen et al. 2008 [89]	79 (40/39)	T: 51.70 ± 4.53 C: 51.67 ± 4.36	Clinical research guiding principles of new medicine of Chinese traditional medicine	Phlegm and blood stasis resistance winding and ascendant hyperactivity of liver yang (79)	Guangzhou
Shen et al. 2005 [90]	290 (120/170)	66.2 ± 1.37	Chinese Guidelines for the Management of Hypertension-2004 (CGMH-2004)	Intense liver fire (34), yin deficiency with yang hyperactivity (99), phlegm-damp retention (114), and deficiency of both yin and yang (43)	Jiangsu
Liu et al. 2009 [91]	240 (120/120)	18–65	Hypertension diagnostic criteria (unclear)	Intense liver fire (240)	Anhui
Lu et al. 2011 [92]	80 (56/24)	T: 66.07 ± 7.15 C: 67.10 ± 7.32	1999 WHO-ISH GMH	Blood stasis (80)	Guangxi
Guo et al. 2006 [93]	60 (30/30)	Not reported	Chinese Guidelines for the Management of Hypertension-2010 (CGMH-2010)	Yin deficiency with yang hyperactivity (60)	Fujian
Zhang et al. 2012 [94]	140 (83/57)	56±10	Chinese Guidelines for the Management of Hypertension-2010 (CGMH-2010)	Ascendant hyperactivity of liver yang (28), yin deficiency with yang hyperactivity (39), phlegm-damp retention (45), and deficiency of both yin and yang (28)	Hebei
Dong et al. 2010 [95]	166 (106/60)	63–82	Hypertension diagnostic criteria (unclear)	Kidney yin deficiency and wind-phlegm (166)	Sichuan

**Table 2:** Number of papers and cases in region.

Region (China)	Provinces	Papers (pieces)	Cases	Male	Female
North China	Hebei	5	552	323	229
	Beijing	6	1374	718	656
	Inner Mongolia	1	53	16	37
	Tianjin	2	247	131	116

Northeast	Liaoning	2	646	376	270
	Heilongjiang	2	152	86	66

Northwest	Xinjiang	2	230	124	106
	Shanxi	1	197	103	94
	Gansu	2	260	148	112

Central China	Henan	2	200	101	99
	Hubei	1	149	74	75
	Hunan	1	120	64	56

East China	Shandong	3	368	211	157
	Jiangsu	10	3004	1489	1515
	Anhui	4	859	421	438
	Zhejiang	6	655	354	301
	Fujian	6	466	307	159
	Jiangxi	1	112	83	29
	Shanghai	3	338	154	184

South China	Guangdong	12	1972	1047	925
	Guangxi	7	936	519	417

Southwest China	Yunnan	1	54	30	24
	Guizhou	2	162	90	72
	Sichuan	1	166	106	60

Total		83	13272	7075	6197

**Table 3:** Syndrome elements of 13,272 patients with essential hypertension.

Syndrome factors	Frequency	Percentage (%)
Yin deficiency	5554	26.27
Yang hyperactivity	4033	19.08
Phlegm turbidity	2892	13.68
Internal fire	2793	13.21
Internal dampness	2333	11.04
Yang deficiency	1668	7.89
Blood stasis	1027	4.86
Qi deficiency	380	1.80
Internal wind	256	1.21
Qi stagnation	164	0.78
Blood deficiency	38	0.18

**Table 4:** Targets of syndrome elements.

Target	Percentage (%)
Liver	7789 (85.68)
Kidney	903 (9.93)
Spleen	399 (4.39)

Total	100

**Table 5:** Combined syndrome forms.

Combination Class	Combination Forms	Frequency	Percentage (%)
Single-factor	Internal fire	2765	20.98
	Yang hyperactivity	875	6.64
	Blood stasis	398	3.02
	Phlegm turbidity	149	1.13

Two-factor	Yin deficiency with yang hyperactivity	3059	23.21
	Phlegm-damp retention	2508	19.03
	Deficiency of both yin and yang	1605	12.18
	Liver-kidney yin deficiency	543	4.12
	Dual deficiency of qi and yin	189	1.43
	Qi stagnation and blood stasis	164	1.24
	Qi deficiency with blood stasis	138	1.05

Three-factor	Yin deficiency and wind-phlegm	166	1.26

Total		12559	95.29

**Table 6:** Chinese herbs and classical formulas that lower BP and improve symptoms according to syndrome differentiation.

Syndrome	Formula	Components	TCM efficacy	Label	Chinese herbs
Internal fire syndrome	*Huanglian Jie Du Tang (detoxicant decoction of Coptis) *	*Rhizoma Coptidis, Radix Scutellariae, Radix et Rhizoma Rhei, and Cortex Phellodendri Chinensis *	Clear heat and toxins from liver	Classical prescription of *Arcane Essentials from the Imperial Library* dispensed by Wang Tao in Tang dynasty	*Xiakucao (Prunella vulgaris *L.*), Huanglian (Coptis chinensis), Huangqin (Scutellaria baicalensis *Georgi*), Huang-bai (Phellodendron *bark*), and Zhizi (Gardenia) *

Yin deficiency with yang hyperactivity	*Tianma Gouteng Yin (decoction of Gastrodia and Uncaria) *	*Rhizoma Gastrodiae, Ramulus Uncariae cum Uncis, Concha Haliotidis, Cortex Eucommiae, Radix Achyranthis Bidentatae, Herba Taxilli, Fructus Gardeniae, Radix Scutellariae, Herba Leonuri, Sclerotium Poriae Pararadicis, and Caulis Polygoni Multiflori *	Suppressing liver yang hyperactivity, clearing heat, activating blood, and nourishing the kidney	Classical prescription of *New Meanings of Treatment in Miscellaneous Diseases with Traditional Chinese Medicine *	*Tianma (Gastrodia), Gouteng (Uncaria), Niuxi (Achyranthes *root*), and Duzhong (Eucommia ulmoides) *

Phlegm-dampness retention	*Wuling powder *	*Alisma, Polyporus, Poria cocos, Ramulus Cinnamomi, Rhizoma Atractylodis Macrocephalae *	Dissolving phlegm, draining water-dampness, and warming Yang	Classical prescription of *Treatise on Febrile and Miscellaneous Diseases* by Zhang Zhongjing in the Eastern Han Dynasty	*Zexie (Alisma), Zhuling (Polyporus), Fuling (Poria cocos), Banxia * (*The tuber of pinellia*),* baizhu (Rhizoma Atractylodis Macrocephalae), Zelan (Herba Lycopi), and Shichangpu (Rhizoma Acori Tatarinowii) *
	*Zexie Tang (decoction of Alisma) *	*Alisma, Rhizoma Atractylodis Macrocephalae *	Dissolving phlegm and draining water-dampness	Classical prescription of *Treatise on Febrile and Miscellaneous Diseases *by Zhang Zhongjing in the Eastern Han Dynasty	
	*Wendan Tang jiawei decoction (modified decoction for clearing away gallbladder heat) *	*Caulis Bambusae in Taenia, Fructus Aurantii Immaturus, Rhizoma Pinelliae, Pericarpium Citri Reticulatae *(aged tangerine peel)*, Poria, Radix et Rhizoma Glycyrrhizae, Radix Codonopsis, Radix Curcumae, and so forth. *	Dissolving phlegm and boosting qi	Modified classical prescription of *Prescriptions Assigned to the Three Categories of Pathogenic * *Factors of Diseases *	

Wind-phlegm	*Banxia Baizhu Tianma Tang (decoction of Pinellia ternata, Atractylodes macrocephala, and Gastrodia elata) *	*Rhizoma Pinelliae Praeparatum, Rhizoma Gastrodiae, Pericarpium Citri Reticulatae, Poria, *and* Radix et Rhizoma Glycyrrhizae *	Calmed the liver, strengthened the spleen, removed dampness, and reduced phlegm	Classical prescription of *Medical Revelations *dispensed by Cheng Zhongling in Qing dynasty	*Fuling (Poria cocos), Banxia (Pinellia ternata), Baizhu (Rhizoma Pinelliae Praeparatum), Tianma (Rhizoma Gastrodiae), and Chenpi (Pericarpium Citri Reticulatae) *

Blood stasis	*Xuefu Zhuyu Tang *	*Radix Angelicae Sinensis, Radix Rehmanniae, Semen Pruni Persicae, red flower* , *Fructus Aurantii, Chinese thorowax root, red peony root, Radix et Rhizoma Glycyrrhizae, Platycodon grandiflorum, Ligusticum chuanxiong *Hort*, and Radix Achyranthis Bidentatae *	Removing blood stasis and promoting Qi	Classical prescription of *Yi Lin Gai Cuo *(correction of the errors of medical works) by Wang Qingren in the Qing Dynasty	*Danggui (Radix Angelicae Sinensis), Chishao (*red peony root*), Danshen (Salvia miltiorrhiza), Yimucao (Leonurus japonicus), Chuanxiong (Ligusticum chuanxiong *Hort*), and Shengdi (Radix Rehmanniae) *

Liver-kidney yin deficiency	*Liu Wei Dihuang Wan (pill of Rehmannia) *	*Rehmannia glutinosa, Fructus corni, Rhizoma Dioscoreae, Alisma, Poria cocos, and Cortex Moutan Radicis *	Replenish liver and kidney yin	*Xiaoer Yaozheng Zhijue *(*Pediatric medicine card straight*) by *Qianyi* in the Song Dynasty	*Shanyurou (Fructus corni), Duzhong (Eucommia), Shudi (Rehmannia glutinosa), Gouqizi * * (Lycium barbarum* L.*), and Huangjing (Rhizoma Polygonati) *

Yang deficiency	*Shen qi Wan *(*kidney qi pill*)	*Rehmannia glutinosa, Fructus corni, Rhizoma Dioscoreae, Alisma, Poria cocos, Cortex Moutan Radicis, Cortex Cinnamomi, and Radix Aconiti Carmichaeli *	Recuperate kidney yang	Classical prescription of *Treatise on Febrile and Miscellaneous Diseases *by Zhang Zhongjing in the Eastern Han Dynasty	*Fuzi (Radix Aconiti Carmichaeli), Bajitian * * (Morinda officinalis),Yinyanghuo (Epimedium), Buguzhi (Psoralea *fruits*), and Rousongrong (Cistanche) *

Qi deficiency	*Buzhong yiqi Tang *	*Codonopsis pilosula, Astragalus membranaceus, Rhizoma Atractylodis Macrocephalae, Tangerine Peel, Rattletop, Radix Bupleuri, Angelica sinensis, and Liquorice *	Replenish qi to invigorate the spleen	Classical prescription of *Treatise on Spleen and Stomach* by Li Dongyuan in the Jin Dynasty	*Dangshen (Codonopsis pilosula), Huangqi (Astragalus membranaceus), and Baizhu (Rhizoma Atractylodis Macrocephalae) *

Blood deficiency	*Danggui siwu Tang *	*Angelica sinensis, Radix Paeoniae Rubra, Ligusticum chuanxiong *Hort*, and Rehmannia glutinosa *Libosch	Enrich and nourish blood	Classical prescription of *Treatise on Febrile and Miscellaneous Diseases *by Zhang Zhongjing in the Eastern Han Dynasty	*Danggui (Angelica sinensis), Chuanxiong (Ligusticum chuanxiong *Hort*), Shudihuang (Rehmannia glutinosa *Libosch*), and Baishao (Radix Paeoniae Rubra) *

## References

[1] Slama M, Susic D, Frohlich ED (2002). Prevention of hypertension. *Current Opinion in Cardiology*.

[2] Calhoun DA, Zaman MA, Nishizaka MK (2002). Resistant hypertension. *Current Hypertension Reports*.

[3] Roger VL, Lloyd-Jones DM, Go AS (2011). Heart disease and stroke statistics 2011 update: a report from the American Heart Association. *Circulation*.

[4] Kearney PM, Whelton M, Reynolds K, Muntner P, Whelton PK, He J (2005). Global burden of hypertension: analysis of worldwide data. *Lancet*.

[5] Lloyd-Jones D, Adams R, Carnethon M (2009). Heart disease and stroke statistics-2009 update: a report from the American Heart Association Statistics Committee and Stroke Statistics Subcommittee. *Circulation*.

[6] Su D, Li L (2011). Trends in the use of complementary and alternative medicine in the United States: 2002–2007. *Journal of Health Care for the Poor and Underserved*.

[7] Ching SM, Zakaria ZA, Paimin F (2013). Complementary alternative medicine use among patients with type 2 diabetes mellitus in the primary care setting: a cross-sectional study in Malaysia. *BMC Complementary and Alternative Medicine*.

[8] Hawk C, Ndetan H, Evans MW (2012). Potential role of complementary and alternative health care providers in chronic disease prevention and health promotion: an analysis of National Health Interview Survey data. *Preventive Medicine*.

[9] Xiong XJ, Yang XC, Liu W (2012). Banxia baizhu tianma decoction for essential hypertension: a systematic review of randomized controlled trials. *Evidence-Based Complementary and Alternative Medicine*.

[10] Xiong XJ, Yang XC, Feng B (2013). Zhen gan xi feng decoction, a traditional Chinese herbal formula, for the treatment of essential hypertension: a systematic review of randomized controlled trials. *Evidence-Based Complementary and Alternative Medicine*.

[11] Wang J, Yang XC, Feng B (2013). Is yangxue qingnao granule combined with antihypertensive drugs, a new integrative medicine therapy, more effective than antihypertensive therapy alone in treating essential hypertension?. *Evidence-Based Complementary and Alternative Medicine*.

[12] Tam WY, Chook P, Qiao M (2013). Cardiovascular protective effects of adjunctive alternative medicine (Salvia miltiorrhiza and Pueraria lobata) in high-risk hypertension. *Evidence-Based Complementary and Alternative Medicine*.

[13] Xu H, Chen KJ (2012). Complementary and alternative medicine: is it possible to be main stream?. *Chinese Journal of Integrative Medicine*.

[14] Ferreira AS, Lopes AJ (2011). Chinese medicine pattern differentiation and its implications for clinical practice. *Chinese Journal of Integrative Medicine*.

[15] Wang J, Xiong XJ, Liu W (2013). Yoga for essential hypertension: a systemic review. *PloS ONE*.

[16] Lee MS, Lim HJ, Lee MS (2004). Impact of qigong exercise on self-efficacy and other cognitive perceptual variables in patients with essential hypertension. *Journal of Alternative and Complementary Medicine*.

[17] Wang J, Feng B, Yang XC (2013). Tai Chi for Essential Hypertension. *Evidence-Based Complementary and Alternative Medicine*.

[18] Wang J, Xiong XJ (2012). Outcome measures of Chinese herbal medicine for hypertension: an overview of systematic reviews. *Evidence-Based Complementary and Alternative Medicine,*.

[19] Xiong XJ, Yang XC, Liu YM (2013). Chinese herbal formulas for treating hypertension in traditional Chinese medicine: perspective of modern science. *Hypertension Research*.

[20] Wang J, Feng B, Yang XC (2013). Tianma gouteng yin as adjunctive treatment for essential hypertension: a systematic review of randomized controlled trials. *Evidence-Based Complementary and Alternative Medicine*.

[21] Wang J, Feng B, Yang XC, Liu W, Xiong X (2013). Chinese herbal medicine for the treatment of prehypertension. *Evidence-Based Complementary and Alternative Medicine*.

[22] Chen KJ (1993). Mao ZD and integrative medicine. *Zhongguo Zhong Xi Yi JieHe Za Zhi*.

[23] Wang J, Yao KW, Yang XC (2012). Chinese patent medicine liu wei di huang wan combined with antihypertensive drugs, a new integrative medicine therapy, for the treatment of essential hypertension: a systematic review of randomized controlled trials. *Evidence-Based Complementary and Alternative Medicine*.

[24] Wang J, Feng B, Xiong XJ (2013). Chinese herbal medicine for the treatment of obesity-related hypertension. *Evidence-Based Complementary and Alternative Medicine*.

[25] Wang J, Xiong XJ (2012). Current situation and perspectives of clinical study in integrative medicine in China. *Evidence-Based Complementary and Alternative Medicine*.

[26] Chen KJ, Jiang YR, Xie YH (2011). Past and present of combination of disease differentiation and syndrome differentiation. *Zhongguo Zhong Xi Yi Jie He Za Zhi*.

[27] Xu H, Chen KJ (2011). Integrating traditional medicine with biomedicine towards a patient-centered healthcare system. *Chinese Journal of Integrative Medicine*.

[28] Chen SL, Liu XY, Xu WM, Mei WY, Chen XL (2012). Clinical study of western medicine combined with Chinese medicine based on syndrome differentiation in the patients with polarized hypertension. *Chinese Journal of Integrative Medicine*.

[29] Liu L, Leung ELH, Tian X (2011). Perspective: the clinical trial barriers. *Nature*.

[30] Dobos G, Tao I (2011). The model of Western Integrative Medicine: the role of Chinese medicine. *Chinese Journal of Integrative Medicine*.

[31] Xiong XJ, Chu FY, Li HX, He QY (2011). Clinical application of the TCM classic formulae for treating chronic bronchitis. *Journal of Traditional Chinese Medicine*.

[32] Wang J, Xiong X (2012). Control strategy on hypertension in Chinese medicine. *Evidence-Based Complementary and Alternative Medicine*.

[33] Wang J, Xiong XJ, Yang GY (2013). Chinese herbal medicine qi ju di huang wan for the treatment of essential hypertension: a systematic review of randomized controlled trials. *Evidence-Based Complementary and Alternative Medicine*.

[34] Tian P (2011). Convergence: where West meets East. *Nature*.

[35] Wang J, Xiong X (2012). Control strategy on hypertension in Chinese medicine. *Evidence-Based Complementary and Alternative Medicine*.

[36] Wang J, Wang PQ, Xiong XJ (2012). Current situation and re-understanding of syndrome and formula syndrome in Chinese medicine. *Internal Medicine*.

[37] Xu H, Chen K (2008). Integrative medicine: the experience from China. *Journal of Alternative and Complementary Medicine*.

[38] Cheung F (2011). TCM: made in China. *Nature*.

[39] Xiong XJ, Yang XC, Liu W (2013). Trends in the treatment of hypertension from the perspective of traditional Chinese medicine. *Evidence-Based Complementary and Alternative Medicine*.

[40] Lu AP, Jia HW, Xiao C, Lu QP (2004). Theory of traditional Chinese medicine and therapeutic method of diseases. *World Journal of Gastroenterology*.

[41] Zhao QG, Li H, Yang HM (2012). Difference of urine protein during the stage of early renal damage in elderly primary hypertensive patients with different yraditional Chinese Medical syndrome types: an analysis of 122 cases. *Journal of Chinese Medicine*.

[42] Liu FM, Chen XH, Du WQ (2009). Correlativity between ACE Gene I/D polymorphism and TCM syndromes of primary hypertension. *Shanghai Journal of Traditional Chinese Medicine*.

[43] Wang XY, Xie ZQ, Ji LL (2012). Clinical research of amlodipine combined Xingqi huoxue decoction on 99 elderly patients with qi stagnation and bood stasis hypertension. *Chinese Medicine Modern Distance Education of China*.

[44] Luo FH, Li GH, Li QL (2011). Clinical research on syndrome differentiation of point application in the treatment of primary hypertension. *China Medical Herald*.

[45] Wang CH, Gao Y, Chen JZ (2012). Effect of syndrome differentiation and treatment combined alisma decoction on traditional Chinese medicine symptom integral of hypertension. *Global Traditional Chinese Medicine*.

[46] Wang YS, Lin ZH, Chen HC (2011). Clinical research on the treatment of early hypertensive renal damage by tonifying kidney and invigorating the circulation of blood. *Traditional Chinese Medicine Journal*.

[47] Bai CJ, Zhou Y, Wang L, Zhang DL, Yang Y (2005). Delamination of cardiovascular risk factor, staging and grading of hypertension and the changing characteristics of blood lipids and hemorheological indexes in hypertensive patients with different syndromes of traditional Chinese medicine. *Chinese Journal of Clinical Rehabilitation*.

[48] Yang LJ, Yao L, Liu AF (2005). Relation study between erythrocyte immunological function and serum SOD activity of hypertension patients with different typing. *Modern Journal of Integrated Traditional Chinese and Western Medicine*.

[49] Xia CX, Yang QY, Zhu HJ (2010). Correlation study on different TCM syndromes of primary hypertension and NO/NOS system, ET21, left ventricular mass index (BMI). *Chinese Journal of Integrative Medicine on Cardio/Cerebrovascular Disease*.

[50] Liu ZY, Wu HL, Luo Y (2003). The effect of shenmai injection dealing with insufficient relaxation of left ventricle caused by hypertension. *Chinese Journal of Chinese Medicine Information*.

[51] Yin WH, Liu JL (2005). Clinical observation on treatment of 53 cases with essential hypertension by deficiency syndrome. *Inner Mongolia Medical Journal*.

[52] Wu R, Zhao FD, Yu SJ (2010). Research of different type of traditional medical syndromes on hypertension disease with regularity of blood pressure variability. *Chinese Journal of Chinese Medicine Emergency*.

[53] Deng HF (2008). Analysis of effect on compound danshen dropping pill compared with sheng tong ping in patients with hypertension. *Asia-Pacific Traditional Medicine*.

[54] Wu LQ, Xu FQ (2010). Study on the relationship between type of syndrome of high-risk hypertensive patients and target organ damage and the risk factors of cardiovascular events. *Chinese Journal of Integrative Medicine on Cardio/Cerebrovascular Disease*.

[55] Wang W, Li FQ, Li YP (2011). Study on the relationship between Syndrome differentiation type of hypertension and Angiotensin original gene M 235T and T 174M polymorphisms. *Journal of Zhejiang Traditional Chinese Medicine*.

[56] Wu DM, Chen XH, Liu FM (2010). Studying of relationship between arteriosclerosis of essential hypertension and TCM syndromes. *Journal of Chinese Medicine in Inner Mongolia*.

[57] Fan QL, Liu FM (2010). Studying of relationship between ambulatory blood pressure of hypertension and syndrome differentiation type of TCM. *Jilin Journal of TCM*.

[58] Zhu W, Li SJ, Ruan XM (2009). Study on the relationship between arterial compliance and syndrome differentiation typing in hypertensive patients. *Lishizhen Medicine and Materia Medica Research*.

[59] Liu FM, Chen XH, Du WQ (2009). Clinical research on the distribution regularity of TCM syndrome types. *Journal of Chinese Medicine in Jiangsu*.

[60] He SY, Fu DY, Zu LH (2013). Correlation of traditional Chinese medicine syndromes with B-type natriuretic peptide and the risk of stroke in patients with essential hypertension: a preliminary study. *China Journal of Traditional Chinese Medicine and Pharmacy*.

[61] Tang JY, Wang YH, Tang N (2012). Correlativity between Patterns of Hypertension in TCM and Polymorphism of COX-2. *Shanghai Zhongyiyao Daxue Xuebao*.

[62] Gong NJ, Li Q, Chen J (2010). A correlated study between TCM syndrome and cardiac structural functional changes in patients with primary hypertension. *Tianjin Journal of Traditional Chinese Medicine*.

[63] Zhang C, Xing ZH, Liu WP (2005). Relativity investigation of plasma endothelin and blood pressure in patients with hypertension with different traditional Chinese medical classification. *Liaoning Journal of TCM*.

[64] Liu FM, Du WQ, Shen L (2009). A correlated study between left ventricular hypertrophy of hypertension and TCM syndrome type. *Liaoning Journal of TCM*.

[65] Wang JY (2012). A correlated study between syndrome differentiation type of TCM and ambulatory blood pressure. *Medical Laboratory Sciences*.

[66] Yao MM, Huang TJ (2007). A correlated study between syndrome differentiation type of TCM and blood uric acid. *Fujian Journal of TCM*.

[67] Guo LL, Zhou Y, Zhuang TT (2002). Relationship between the endothelin/nitric oxide and hypertension with traditional Chinese medical classification. *Guizhou Medical Journal*.

[68] Zhang JZ, Chen LG, Hu XQ (2011). Influence of astragalus polysaccharide on the expression of Toll-like receptor 4 and nuclear transcription factor-k B in essential hypertension patients with blood stasis syndrome. *Journal of Traditional Chinese Medicine*.

[69] Liao WQ, Huang WM, He B (2010). Observation on insulin resistance of essential hypertension treated by the therapeutic method of activating blood circulation to dissipate Blood Stasis. *Chinese Journal of Information on TCM*.

[70] Xiong YW (2010). Clinical effect of the modified banxia baizhu tianma decoction combining western medicine on 60 patients with phlegm-dampness type primary hypertension. *Chinese Medicine Modern Distance Education of China*.

[71] Jiang CX, Cao JM, Xu JY (2012). Clinical research on effects of the renin-angiotensin-II of senile patients with high blood pressure by jianling decoction. *Chinese Journal of Science and Technology of Chinese Medicine*.

[72] Huang ZC, Wei QP (2012). Curative effect analysis on identifying treatment of resistant hypertension combined with the system of traditional Chinese medicine. *Chinese Community Doctors*.

[73] Lu X (2004). Clinical research on the treatment of patients with liver-kidney yin deficiency of hypertension by qiju dihuang wan decoction. *Xinjiang Journal of Chinese Medicine*.

[74] Sun HT, Wang C (2005). Different manifestations of cognitive dysfunction in hypertensive patients with different syndromes of traditional Chinese medicine. *Chinese Journal of Clinical Rehabilitation*.

[75] Xiang Y, Bai GC, Wu LM (2012). Syndrome differentiation type and of nursing modern of community of primary hypertension. *Hebei Journal of TCM*.

[76] Zhu DX (2009). The effect of injected Ligustrazine to shenshu point in patients with hypertension. *China Practical Medicine*.

[77] Xu CY, Wang FL (2009). Clinical observation of hypertensive effect of taichong acupoint injection with ligustrazine on hypertension. *Occupations and Health*.

[78] Lin M, Kang NS (2012). Study on carotid ultrasound of phlegm dampness syndromes compared with yang hyperactivity syndromes of not intervention in primary hypertension. *Journal of Shandong University of TCM*.

[79] Feng H, Liu ZC, Xu B (2013). Clinical observation on the treatment of 36 cases of excessive accumulation of phlegm-dampness of essential hypertension complicated by obesity by warm acupuncture. *Journal of Anhui TCM College*.

[80] Yu AW, Xing EH (2010). Clinical observation on the treatment of 90 cases with hypertension by Xifeng Tongluo Huayu decoction. *Journal of Sichuan of TCM*.

[81] Qiu C, Cheng XD, Cheng FK (2011). Clinical observation on the treatment of senile hypertension by Yiqihuoxue decoction. *Hebei Journal of TCM*.

[82] Wu TC, Xu T (2012). Clinical observation on the treatment of 60 cases of senile hypertension by tonifying qi and yin. *Yunnan Journal of TCM*.

[83] Fang XM, Huang XY, Wang Q (2007). Research of clustering analysis of syndrome differentiation type of hypertension. *Guangxi Journal of TCM*.

[84] Fang W, Chen TL, Zhu GL (2003). Research on the syndrome differentiation type of primary hypertension and the characteristics of dynamic blood pressure changes. *Zhejiang Journal of TCM*.

[85] Peng LL, Shi DZ (2010). Analysis on syndromes elements of primary hypertension with depression. *Beijing Journal of TCM*.

[86] Yang HY, Jin YR, Yang H (2004). Relationship between the syndrome differentiation type of hypertension and ambulatory blood pressure. *Chinese Journal of Information TCM*.

[87] Shi XL, Wen ZL, Zhang N (2013). Clinical investigation on related factors and rule of the type of distribution of primary hypertension with depression. *Jiangsu Journal of TCM*.

[88] Han YM (2004). Clinical study on the treatment of primary hypertension based on syndrome differentiation of TCM. *Health Guides of the International Medical*.

[89] Shen R, Chen YD, Zhang ZX (2008). Exploring the correlation of syndrome differentiation of primary hypertension and heart rate variability. *China Journal of Traditional Chinese Medicine and Pharmacy*.

[90] Shen Y, Zhang JD, Hu LH (2005). The relatirity of insulin resistance and syndrome differentiation typing in essential hypertension. *Journal of Shandong University*.

[91] Liu XL, Wei AL, Luo F (2009). Ultrasound study of carotid artery plaque score and the carotid intinamedia thickness in essential hypertension with traditional chinese medical classification. *Liaoning Journal of TCM*.

[92] Lu X, Wei CY, Yang LH (2011). Study on the relationship between syndrome type of TCM of primary hypertension and apolipoprotein. *Journal of Guangxi Traditional Chinese Medical University*.

[93] Guo KF, Zhang JF, Yang WQ (2006). Relationships between personality characteristic and diff erentiation of symptom and sign for classification of syndrome of traditional Chinese medicine in patients with primary hypertension. *Chinese Journal of Cardiovascular Rehabilitation Medicine*.

[94] Zhang JP, Yuan HW, Wang HL (2012). Correlation between concentration of *β*2 microglobulin in blood and urine in patients with primary hypertension and traditional Chinese medicine syndrome. *Hebei Journal of TCM*.

[95] Dong CW, Dong M, Xing QS (2010). Relationship between polymorphism of angiotensin converting enzyme gene and different traditional Chinese medicine syndrome in patients with essential hypertension. *Chinese Journal of Pathophysiology*.

[96] Zhang ZB, Zhou CG, Lu S (2010). Distribution of TCM syndrome types of essential hypertension and their relationship to biochemical indicators. *Liaoning Journal of TCM*.

[97] Chen J, Chen ZQ (2008). Clinical study on characteristics of changes in dynamic blood pressure in patients with primary hypertension of blood stasis syndrome. *World Journal of Integrated Traditional and Western Medicine*.

[98] Liu L, Zhang YQ (2010). A correlated study between HCY, MAU and TCM syndrome type of hypertension. *Heilongjiang Journal of TCM*.

[99] Zhou X (2013). Clinical effect analysis on treating 86 cases of essential hypertension with treatment based on differentiation of symptoms and signs. *Clinical Journal of Chinese Medicine*.

[100] Chen GY, Wang LJ, Liu J (2012). Relationship between TCM syndrome types and related risk factors of essential hypertension. *Chinese Journal of Information on TCM*.

[101] Zhou WQ, Liu DH, Dai YN (2009). Study on the correlation between the related factors and TCM syndromes of essential hypertension with left ventricular hypertrophy. *Journal of Traditional Chinese Medicine*.

[102] Ding YX, Zhou YX, Liu B (2009). Preliminary analysis on epidemiological characteristics of TCM syndromes of primary hypertension in Guangdong region. *Journal of Anhui TCM College*.

[103] Li ZW (2002). Clinical observation on the treatment of 125 cases of essential hypertension complicated by syndrome differentiation and treatment of TCM. *Forum on Traditional Chinese Medicine*.

[104] Qiu ZF (2010). Clinical observation on the treatment of 97 cases of essential hypertension by syndrome differentiation of TCM. *Chinese Journal of Ethnomedicine and Ethnopharmacy*.

[105] Deng WL, Zhao G (2004). Clinical observation on syndrome differentiation of TCM of 80 cases of the hypertension patients with left ventricular hypertrophy. *Xinjiang Journal of TCM*.

[106] Chen Q (2010). Clinical study on primary hypertension early time kidney harm with Zishuiqingganyin. *Chinese Archives of TCM*.

[107] Zhang TW, Zheng BR (2009). Clinical observation on the treatment of 30 cases of essential hypertension with liver-yang hyperactivity by “ Jiangya 2”. *Fujian Medical Journal*.

[108] Wan YX, Zhang TZ (2008). Research on relationship between angiotensinogen gene M235T and TCM syndrome type in essential hypertension patients. *Chinese Journal of Integrated Traditional and Western Medicine*.

[109] Xu YJ, Zhang TZ (2008). Relationship of day and night rhythm of Essential Hypertension with TCM Syndrome patterns. *Journal of New Chinese Medicine*.

[110] Zhang D, Li HR (2010). Epidemiological investigation and distribution of TCM syndrome of primary hypertension in community. *The Community of Chinese Medicine*.

[111] Pu ZH, Yang GD, Ding TY (2008). Clinical observation on the effect of blood pressure, cholesterol, blood sugar, and the clinical syndromes of traditional Chinese medicine of 220 cases of essential hypertension by “Ping ganyin” capsule. *China Journal of Chinese Materia Medica*.

[112] Cheng WJ, Tan ZC, Guo F (2003). Epidemiological studies on TCM syndrome of 602 cases of primary hypertension. *Chinese Journal of Integrated Traditional and Western Medicine*.

[113] Ding BY, Shao ZB, Zheng L (2006). Study on the relationship between early nephritic injures by essential hypertension and traditional Chinese medicine syndrome patterns. *Journal of Anhui TCM College*.

[114] Fu S, Li YL (2010). Establishing standards of measuring essential hypertension with hyperactive of liver-Yang syndrome based on multivariate analysis of statistics. *Journal of Shandong University of TCM*.

[115] Pan Q, Liu ZD, Chen LP (2012). Shiliangcha Prescription of “She medicine” combined with western medicine in treating essential hypertension of damp-phlegm pattern: a report of 30 cases. *Shanghai Journal of TCM*.

[116] Chen GL, Wang BY, Liu HP (2011). Survey on traditional Chinese medicine syndrome types in 471 patients with essential hypertension. *Journal of Anhui TCM College*.

[117] Zhao YH, Liu YD, Huang PG (2009). Effects of clinical treatment and protection on endothelial function of early essential hypertension’s patients treated with YNJY soup in southern Guangdong area. *China Journal of Traditional Chinese Medicine and Pharmacy*.

[118] Zhou CG, Zhang ZB, Xia CX (2010). Association of the C-344T polymorphism of CY P11 B2 gene with TCM syndrome type in essential hypertension. *Liaoning Journal of TCM*.

[119] Huang JH, Zheng QS, Gao R (2004). Clinical equivalence evaluation on the efficacy and safety of Niuhuang jiangya tablets and pills in the treatment of patients with primary hypertension (overabundant liver-fire). *Chinese Journal of Evidence-Based Medicine*.

[120] Lu X (2004). Clinical observation on the treatment of essential hypertension by compound Danshen dripping pills. *Journal of Guangxi Traditional Chinese Medical University*.

[121] Zhu D, Lin SB (2012). Clinical observation on the treatment of essential hypertension with yin deficiency and yang excess by Zishui pinggan decoction. *Chinese Journal of Geriatric Care*.

[122] Wang CH, Gao Y, Chen JZ (2013). Impact analysis of syndrome differentiation combined with modified Zixie decoction for hypertension symptoms. *World Chinese Medicine*.

[123] Ji DG (2011). Ling kok uncaria decoction in the treatment of hypertension. *Journal of Chinese Herbs of TCM*.

[124] Yuan L, Tu D, Ye X, Wu J (2006). Hypoglycemic and hypocholesterolemic effects of Coptis chinensis franch inflorescence. *Plant Foods for Human Nutrition*.

[125] Koo HJ, Lim KH, Jung HJ, Park EH (2006). Anti-inflammatory evaluation of gardenia extract, geniposide and genipin. *Journal of Ethnopharmacology*.

[126] Ho SC, Ho YF, Lai TH, Liu TH, Wu RY (2005). Traditional Chinese herbs against hypertension enhance the effect of memory acquisition. *American Journal of Chinese Medicine*.

[127] Zhou J, Zhou S (2010). Antihypertensive and neuroprotective activities of rhynchophylline: the role of rhynchophylline in neurotransmission and ion channel activity. *Journal of Ethnopharmacology*.

[128] Hansen K, Nyman U, Smitt UW (1995). In vitro screening of traditional medicines for anti-hypertensive effect based on inhibition of the angiotensin converting enzyme (ACE). *Journal of Ethnopharmacology*.

[129] Gu J, Wang JJ, Yan J (2011). Effects of lignans extracted from Eucommia ulmoides and aldose reductase inhibitor epalrestat on hypertensive vascular remodeling. *Journal of Ethnopharmacology*.

[130] Li L, Yan J, Hu K (2012). Protective effects of Eucommia lignans against hypertensive renal injury by inhibiting expression of aldose reductase. *Journal of Ethnopharmacology*.

[131] Greenway F, Liu Z, Yu Y, Gupta A (2011). A clinical trial testing the safety and efficacy of a standardized Eucommia ulmoides oliver bark extract to treat hypertension. *Alternative Medicine Review*.

[132] Han YP, Wang NS, Mi SQ, Liu QD (2003). Effect of Wuling Powder on rats with renal hypertension. *Zhong Xi Yi Jie He Xue Bao*.

[133] Chen JY, Fan HL, Zhang SF (2012). Effect of modified Zexie decoction on prevention of kidney injuries of rats with hypertension induced by high-salt diet. *Zhong Yi Za Zhi*.

[134] Wu SJ, Ng LT, Lin CC (2004). Antioxidant activities of some common ingredients of traditional Chinese medicine, Angelica sinensis, Lycium barbarum and Poria cocos. *Phytotherapy Research*.

[135] Sun Y, Wang S, Li T, Li X, Jiao L, Zhang L (2008). Purification, structure and immunobiological activity of a new water-soluble polysaccharide from the mycelium of Polyporus albicans (Imaz.) Teng. *Bioresource Technology*.

[136] Yuan R, Lin Y (2000). Traditional Chinese medicinean approach to scientific proof and clinical validation. *Pharmacology and Therapeutics*.

[137] Jiang WY (2005). Therapeutic wisdom in traditional Chinese medicine: a perspective from modern science. *Trends in Pharmacological Sciences*.

[138] Kim DD, Sánchez FA, Durán RG, Kanetaka T, Durán WN (2007). Endothelial nitric oxide synthase is a molecular vascular target for the Chinese herb Danshen in hypertension. *American Journal of Physiology—Heart and Circulatory Physiology*.

[139] Hou YZ, Zhao GR, Yuan YJ, Zhu GG, Hiltunen R (2005). Inhibition of rat vascular smooth muscle cell proliferation by extract of Ligusticum chuanxiong and Angelica sinensis. *Journal of Ethnopharmacology*.

[140] Simeonova RL, Vitcheva VB, Kondeva-Burdina MS, Krasteva IN, Nikolov SD, Mitcheva MK (2010). Effect of purified saponin mixture from Astragalus corniculatus on enzyme- and non-enzyme-induced lipid peroxidation in liver microsomes from spontaneously hypertensive rats and normotensive rats. *Phytomedicine*.

[141] Xue B, Li J, Chai Q, Liu Z, Chen L (2008). Effect of total flavonoid fraction of Astragalus complanatus R. Brown on angiotensin II-induced portal-vein contraction in hypertensive rats. *Phytomedicine*.

[142] Yao LM, Liu TW, Wu WF (2009). Effects of Astragalus injection in reversing left ventricular hypertrophy induced by renal hypertension in rats. *Zhongguo Zhong Xi Yi Jie He Za Zhi*.

[143] Lin LZ, He XG, Lian LZ, King W, Elliott J (1998). Liquid chromatographic-electrospray mass spectrometric study of the phthalides of Angelica sinensis and chemical changes of Z-ligustilide. *Journal of Chromatography A*.

[144] Hou YZ, Zhao GR, Yang J, Yuan YJ, Zhu GG, Hiltunen R (2004). Protective effect of Ligusticum chuanxiong and Angelica sinensis on endothelial cell damage induced by hydrogen peroxide. *Life Sciences*.

